# From bench to bedside: therapeutic potential of natural antioxidants in diabetic neuropathy

**DOI:** 10.3389/fcell.2025.1631678

**Published:** 2025-10-03

**Authors:** Yanxian Zhang, Lili Yao, Yongmei Lyu, Zhuqi Tang, Xiaoyu Liu, Xuchu Duan

**Affiliations:** ^1^ School of Life Sciences, Department of Endocrinology, Key Laboratory of Neuroregeneration of Jiangsu and Ministry of Education, Co-Innovation Center of Neuroregeneration, Affiliated Hospital of Nantong University, Nantong University, Nantong, China; ^2^ School of Marine and Bioengineering, Yancheng Institute of Technology, Yancheng, China

**Keywords:** diabetic neuropathy, oxidative stress, natural antioxidants, mechanisms, treatments

## Abstract

Diabetic neuropathy (DN) is a prevalent and debilitating complication of diabetes, causing substantial morbidity and negatively impacting the quality of life for millions of individuals worldwide. The pathogenesis of DN is complex, with oxidative stress (OS) emerging as a key factor contributing to nerve damage through mechanisms like lipid peroxidation, protein modification, and DNA damage. This review examines the role of natural antioxidants in alleviating symptoms of DN, summarizes recent progress in fundamental and clinical research on antioxidants in treating DN. It emphasizes the mechanisms by which compounds such as polyphenols, terpenoids, and carotenoids counteract OS, a critical factor in the pathogenesis of DN. These antioxidants, derived from various natural sources, show promise in enhancing nerve conduction velocity, reducing neuropathic pain, and improving glucose metabolism. Clinical trials, particularly those involving alpha-lipoic acid, provide evidence supporting the benefits of antioxidant therapy in enhancing nerve function. The review highlights the necessity for further research into natural antioxidants to develop more effective treatment strategies for DN.

## 1 Introduction

### 1.1 Diabetic neuropathy

Diabetic neuropathy (DN) is a significant complication of diabetes mellitus, characterized by nerve damage resulting from prolonged hyperglycemia. This condition affects 60%–70% of diabetic patients may experience some form of neuropathy during their lifetime. The progression of DN can lead to severe consequences, including pain, loss of sensation, and increased risk of foot ulcers and amputations, which collectively strain global healthcare resources (Bondar et al., 2021; Anumah et al., 2022). Even with good glycemic control, the prevalence of DN in individuals still ranges from approximately 10%–30% (Abbott et al., 2011). Therefore, regular screening and management of DN are essential for individuals with diabetes.

The clinical manifestations of DN can vary widely, ranging from asymptomatic cases to severe pain and functional impairment. For instance, patients may experience symptoms such as numbness, tingling, and burning sensations, which can significantly impact their quality of life (Won et al., 2017; Huang et al., 2023). Moreover, the relationship between DN and other complications, such as cardiovascular autonomic neuropathy, has been established, indicating that these conditions often coexist and may share common pathophysiological mechanisms (de Paula et al., 2024; Braffett et al., 2020).

The impact of DN extends beyond the individual, affecting public health systems due to the associated morbidity and mortality. Diabetic peripheral neuropathy (DPN) is a leading cause of lower-limb amputation, which significantly diminishes life expectancy and quality of life (Selvarajah et al., 2019). Furthermore, the psychological burden of chronic pain and disability associated with neuropathy can lead to increased rates of anxiety and depression among affected individuals (Zhang et al., 2019). Given the multifaceted nature of DN and its widespread implications, it is crucial for healthcare providers to recognize and address this condition through early diagnosis, effective pain management, and comprehensive care strategies aimed at improving patient outcomes and reducing the overall burden on public health systems (Iqbal et al., 2018; Mohseni et al., 2021).

### 1.2 Pathophysiology and mechanisms of DN

DN is a significant microvascular complication of diabetes mellitus, characterized by a range of nerve dysfunctions that can lead to debilitating symptoms such as pain, numbness, and other neurological impairments. The pathophysiology of DN is complex and multifactorial, involving metabolic, vascular, and inflammatory mechanisms that converge to damage peripheral nerves. Understanding these mechanisms is essential for the development of effective therapeutic strategies to manage and potentially prevent this debilitating condition.

The first significant factor in the pathogenesis of DN is glycemic variability and hyperglycemia. Glycemic variability refers to fluctuations in blood glucose levels that can contribute to the development of diabetic complications. Recent studies have suggested that glycemic variability may serve as an independent risk factor for DN, affecting both peripheral and autonomic nerve functions (Zhang X. et al., 2021). This variability can cause repeated episodes of nerve ischemia and reperfusion, leading to further oxidative damage and inflammatory responses. Hyperglycemia plays a critical role in the development of diabetic complications, particularly through the formation of advanced glycation end products (AGEs), which further exacerbate oxidative stress (OS) and promote inflammation within the nervous system (Sivakumar et al., 2022; Mengstie et al., 2022). Accumulation of AGEs is linked to the pathogenesis of DN, where they exacerbate microvascular complications and lead to nerve degeneration (Zhang X. et al., 2021). Additionally, hyperglycemia is associated with decreased nerve fiber diameter, reduced number of large myelinated fibers, and decreased nerve conduction velocity, which are more pronounced in cases of early-onset hyperglycemia (Strand et al., 2024).

Inflammation also plays a crucial role in the development and progression of DN. Low-grade intraneural inflammation has been observed in DN, which is associated with the degeneration of intraepidermal nerve fibers. This inflammatory process may be triggered by obesity and dyslipidemia, even in the absence of overt diabetes mellitus (Baum et al., 2021). Recent studies have also highlighted the role of neuroinflammation and the malfunctioning of glial cells in DN. High glucose and OS-mediated damage in neurons and glial cells, along with neuroinflammation, are essential mechanisms underlying the progression of DN (Rahman et al., 2016).

A further driver of nerve injury is heightened OS, which has been implicated in the progression of nerve injury in diabetic patients, as it can lead to cellular damage and apoptosis of neurons and Schwann cells (Pang et al., 2020). OS is characterized by a disequilibrium between the generation of reactive oxygen species (ROS) and the organism’s capacity to neutralize these reactive intermediates or rectify the resultant damage. This disequilibrium can result in cellular injury and is implicated in the pathogenesis of neuropathy, wherein nerve damage arises from sustained OS. In DN, persistent hyperglycemia is known to augment OS, thereby contributing to nerve damage and dysfunction. Empirical studies have demonstrated that markers of OS, such as malondialdehyde and protein carbonyls, are elevated in individuals with diabetes and DN, signifying increased lipid peroxidation and protein oxidation (Almogbel et al., 2017). Furthermore, the activation of NADPH oxidases, particularly NOX4, has been identified as a significant source of ROS in diabetic contexts, thereby intensifying oxidative damage to neural tissues (Syed et al., 2023).

OS is closely associated with neuropathy. This review offers a comprehensive synthesis of recent advancements in the investigation of antioxidants for the treatment of DN, with a particular focus on their clinical application, molecular mechanisms and potential therapeutic applications.

## 2 Mechanisms of OS-induced nerve damage

**FIGURE 1:**
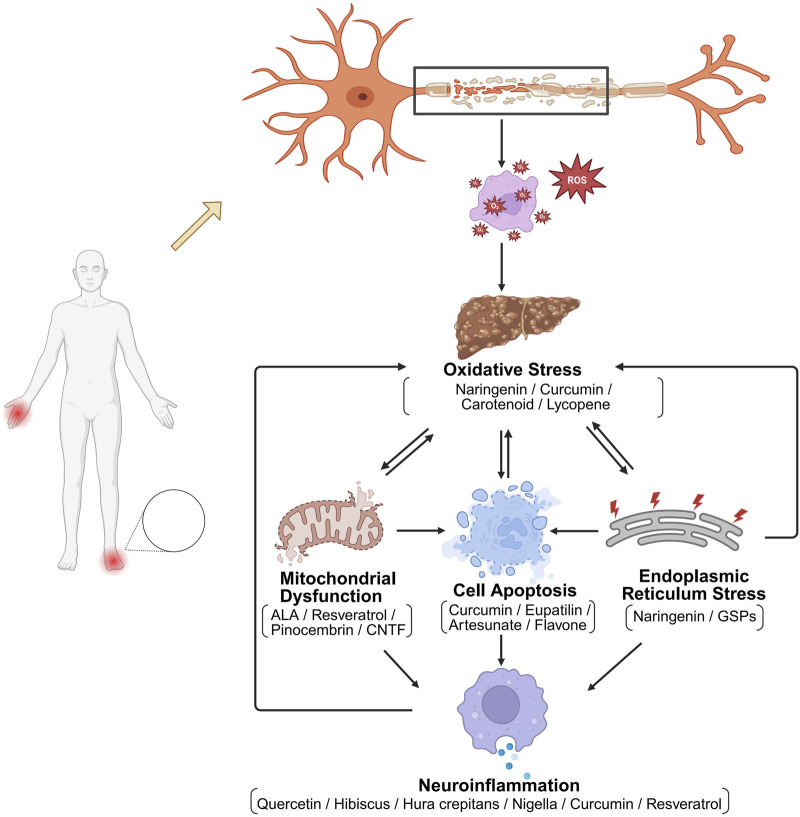
Mechanisms of OS-induced nerve damage. Simplified scheme of OS–triggered cellular damage in diabetic neuropathy and representative natural antioxidants (Naringenin, Curcumin, Carotenoids, Lycopene, Nigella, etc.) that counteract mitochondrial/ER stress-mediated apoptosis and neuroinflammation.

In the context of DN, OS contributes to oxidase activation, mitochondrial dysfunction, apoptosis, inflammation, and endoplasmic reticulum (ER) stress, which are critical in the pathogenesis of nerve injury (Figure 1). One of the key mechanisms involve the activation of NADPH oxidases, which are enzymes that generate ROS (Zhao et al., 2014). Increased ROS levels can lead to lipid peroxidation, protein modification, and DNA damage, exacerbating neuronal injury. Moreover, OS can activate various signaling pathways, including the NF-κB pathway, which promotes the expression of pro-inflammatory cytokines such as TNF-α and IL-6, further contributing to neuroinflammation and neuronal apoptosis (Kumar et al., 2012; Sharan et al., 2024). The interplay between OS and inflammation is crucial in understanding neuropathy. Inflammatory cytokines can amplify OS, creating a vicious cycle that leads to further nerve injury. The inhibition of inflammatory pathways reduces OS and improve neuropathic symptoms in experimental models (Negi et al., 2011a). This suggests the potential therapeutic strategies targeting both OS and inflammation to mitigate neuropathy.

Mitochondrial dysfunction is another significant aspect of OS in DN (Chen and Fang, 2018). Hyperglycemia-induced OS can impair mitochondrial function, leading to decreased ATP production and increased ROS generation. This vicious cycle of OS and mitochondrial dysfunction is associated with the activation of apoptotic pathways, including the upregulation of pro-apoptotic proteins like Bax and the downregulation of anti-apoptotic proteins like Bcl-2 (Zhu et al., 2023; Zhu et al., 2018).

Recent studies have also highlighted the role of ER stress in the context of OS and DN. The accumulation of misfolded proteins in the ER can trigger a stress response that leads to increased ROS production and inflammation, contributing to neuronal cell death (Fan et al., 2017; Pandey et al., 2019). Moreover, OS affects the integrity of the blood-nerve barrier (BNB), leading to increased permeability and subsequent infiltration of inflammatory cells into the nerve tissue. This process further exacerbates oxidative damage and neuronal injury (Burgos-Morón et al., 2019).

As a pivotal driver of neuronal damage and disease progression, OS also regulates various intracellular signaling pathways. The phosphoinositide 3-kinase (PI3K), Akt, and mammalian target of rapamycin (mTOR) signaling pathways have been identified as significant players in mediating the effects of OS in the nervous system (Maiese et al., 2012). The AMPK/ERK1/2 signaling pathway has been identified as a critical mediator of OS-induced neuronal damage in cerebral ischemia-reperfusion injury (Zhang et al., 2024). In spinal cord injury, OS is a major pathological event that contributes to secondary injury and neurological dysfunction. The nuclear factor erythrocyte 2-associated factor 2 (Nrf2) and NF-κB signaling pathway have been identified as a critical regulator of antioxidant responses in spinal cord injury (Xiao et al., 2024; Yang et al., 2018). Activation of the Nrf2 pathway in Schwann cells can promote the recovery of nerve function by reducing OS and inflammation, which are critical components of secondary injury following sciatic nerve injury (Zhou et al., 2023). Furthermore, the impact of OS on intracellular signaling pathways such as the mitogen-activated protein kinase (MAPK) and c-Jun N-terminal kinase (JNK) pathways were also validated. These pathways are activated in response to OS, leading to apoptosis and inflammation, further contributing to neurodegeneration. The modulation of these signaling pathways through therapeutic interventions, such as the use of phytochemicals or specific inhibitors, holds promise for mitigating the harmful effects of OS and improving outcomes in neurodegenerative diseases (Sebghatollahi et al., 2025).

## 3 Different natural antioxidative compounds in DN treatments

Natural antioxidants neutralise free radicals and thereby lower the risk of chronic diseases such as cancer and cardiovascular disorders. Key bioactive compounds—polyphenols, flavonoids, and vitamins C and E—are found in various plant-based sources, working synergistically to strengthen cellular defenses, reduce inflammation, and delay aging. These antioxidants often exhibit higher safety and bioavailability compared to synthetic alternatives, enhancing their use in functional foods, supplements, and cosmetics. Ongoing research focuses on elucidating their molecular mechanisms and optimizing health applications, emphasizing their potential in preventive medicine and holistic wellness strategies (Tables 1, 2).

**TABLE 1:** Overview of natural antioxidants discussed in this review.

Class	Representative compound (key structural motif)	Principal natural origin(s)	Highest level of evidence	Key mechanism(s) highlighted in DN context
Polyphenols	Quercetin (flavonol, 3-OH, 5-OH, 7-OH)	Onion skin, apples, tea leaves	Animal (STZ rat) + small RCT (combo with gliclazide)	Nrf2↑, NF-κB↓, inflammasome inhibition
	Curcumin (diarylheptanoid, β-diketone)	Curcuma longa rhizome	RCT (nano-curcumin, n = 40)	NOX4↓, NGF↑, PI3K/Akt/mTOR
	Naringenin (flavanone, 4′,5,7-triOH)	Citrus peels	Animal	TNF-α↓, oxidative-nitrosative stress↓
	Luteolin (flavone, 3′,4′,5,7-tetraOH)	Chrysanthemum, carrot leaves	Animal	Nrf2/HO-1↑
	Rutin (quercetin-3-O-rutinoside)	Buckwheat, Sophora japonica	Animal	Nrf2↑, plasma glucose↓
Terpenoids	Eugenol (allyl-substituted guaiacol)	Syzygium aromaticum bud oil	Animal	PPAR-α↑, lipid peroxidation↓
	Trans-anethole (propenyl-substituted anisole)	Foeniculum vulgare seed	Animal	Carbohydrate-metabolising enzyme modulation
	Astaxanthin (xanthophyll, conjugated polyene)	Haematococcus pluvialis, shrimp shell	Animal	Mitochondrial cardiolipin stabilisation, ROS↓
Carotenoids	Lycopene (acyclic carotene, 11 conjugated double bonds)	Tomato, watermelon	Animal	AGE detoxification↑, antioxidant defence↑
	β-Carotene (cyclic carotene)	Carrot, palm oil	Epidemiology (Chinese cohort)	Diabetes risk↓
Vitamins/Co-factors	α-Lipoic acid (octanoic acid with 1,2-dithiolane)	Endogenous; spinach, red meat	Multiple RCTs (n ≈ 1800)	Redox cycling, Nrf2↑, NF-κB↓
	Vitamin E (α-tocopherol, chromanol ring)	Wheat-germ oil, nuts	Meta-analysis of RCTs	Paraoxonase-1↑, NCV improvement
	Vitamin B12 (methylcobalamin)	Animal liver, dairy	RCT (n = 90, 12 months)	Myelin repair, NCV↑

**TABLE 2:** Description of the antioxidant natural products and pharmacological aspects of the preclinical studies included in the review.

Substance	Animal model (sex, n)	Dose & route	Duration	NCV (m s^−1^, Δ vs. control)	MDA (nmol mg^−1^, %↓)	SOD (U mg^−1^, %↑)	TNF-α (pg mg^−1^, %↓)	Apoptosis (%↓ vs. control)	Ref
Quercetin	C57BL/6, n = 10	100 mg kg^−1^ i.p.	7 days	+2.3	−38%	+42%	—	−35%	Domiciano et al. (2017)
Hibiscus polyphenols	SH-SY5Y cells	10 μM	24 h	—	−45%	+55%	−40%	—	Zhang et al. (2020)
Naringin	Wistar SD, n = 12	40 mg kg^−1^ p.o.	4 weeks	+2.1	−30%	+38%	−33%	−28%	Ahmad et al. (2022)
Lycopene	Wistar SD, n = 10	4 mg kg^−1^ p.o.	7 days	+1.8	−42%	+35%	−36%	−31%	Celik et al. (2020)
Curcumin	SD, n = 12	150 mg kg^−1^ p.o.	4 weeks	+3.2	−50%	+45%	−52%	−40%	Zhang et al. (2022)
Eugenol	Wistar SD, n = 10	24 mg kg^−1^ i.p.	4 weeks	+1.9	−35%	+30%	−29%	−25%	Kokabiyan et al. (2023)
Trans-anethole	Wistar SD, n = 10	80 mg kg^−1^ p.o.	45 days	+2.0	−28%	+32%	—	−22%	Sheikh et al. (2015)
Terpenoids (Hura crepitans)	Wistar SD, n = 8	300 mg kg^−1^ p.o.	2 weeks	+2.2	−40%	+41%	−30%	−27%	Lukman et al. (2024)
Rutin	Wistar SD, n = 12	50 mg kg^−1^ p.o.	2 weeks	+2.4	−43%	+48%	−38%	−33%	Tian et al. (2016)
Astaxanthin	Wistar SD, n = 10	50 mg kg^−1^ p.o.	24 h	+1.6	−32%	+29%	−25%	−20%	Ciapała et al. (2024)
Luteolin	Wistar SD, n = 12	200 mg kg^−1^ p.o.	3 weeks	+2.5	—	—	—	—	Domiciano et al. (2017)

Balb/c, Bagg albino c mouse strain; Wistar SD, Wistar Sprague-Dawley rat strain; NCV, nerve conduction velocity; MDA, malondialdehyde.

### 3.1 Polyphenols

Polyphenols, which are abundant in various fruits, vegetables, and beverages, exhibit strong antioxidant properties, have garnered significant attention in the treatment of DN because their multifaceted roles in mitigating OS and inflammation associated with diabetes. This group includes flavonoids, phenolic acids, and tannins, which are widely recognized for their antioxidant capacity. Quercetin is a kind of flavonoid, which could reduce blood glucose levels and improve lipid profiles, inhibit inflammasome activation and reduce OS, demonstrating its potential as a therapeutic agent in inflammatory diseases (Domiciano et al., 2017; Ahmad et al., 2017). Additionally, polyphenols from the flower of *Hibiscus syriacus* have been reported to ameliorate neuroinflammation, exerting neuroprotective effects (Zhang et al., 2020). Naringenin ameliorates DN pain by modulating oxidative-nitrosative stress and inflammatory cytokines, thereby improving nerve conduction velocity and reducing pain perception in diabetic rats (Kandhare et al., 2012). Additionally, the flavonoid-rich fraction of *Tephrosia purpurea* was reported to ameliorate hyperglycemia and cardiovascular complications associated with diabetes (Bhadada and Goya, 2016).

Herbal products are rich in various active antioxidant compounds. A promising herbal compound is luteolin, which improves nerve functions in DN through its antioxidant and neuroprotective effects. Luteolin administration in diabetic rats resulted in improved nerve conduction velocities and reduced OS markers (Li M. et al., 2015). Rutin is a flavonoid found in various plants, has been reported to ameliorate DN by lowering plasma glucose levels and decreasing OS via the Nrf2 signaling pathway (Tian et al., 2016). Another significant compound is Eupatilin, a flavonoid extracted from *Artemisia*, which has demonstrated the ability to induce apoptosis in various cancer types and has been investigated for its effects on colon cancer cells. Its mechanisms include the induction of OS and modulation of signaling pathways, which may also be relevant in neuropathic pain management (Lee et al., 2021). Sesamol, derived from sesame, exhibits strong antioxidant properties and has been studied for its derivatives, which also show significant antioxidant activity (Zhou et al., 2021; Chopra et al., 2010).

The protective effects of polyphenols extend beyond just antioxidant activity, they also enhance neurotrophic support, which is crucial for neuronal survival and function. For example, curcumin has been reported to prevent diabetic retinopathy through its hypoglycemic, antioxidant, and anti-inflammatory mechanisms (Gupta et al., 2011; Gutierres et al., 2019). It was also found to ameliorate DN by inhibiting NADPH oxidase-mediated OS in the spinal cord (Zhao et al., 2014). A recent study explored curcumin’s effects on STZ-induced DN in rats and found that curcumin improved nerve function, reduced Schwann cell apoptosis, and increased NGF levels in sciatic nerves and serum (Zhang et al., 2022). Unlike other polyphenols, curcumin simultaneously inhibits NOX4-mediated superoxide burst and up-regulates NGF, providing a dual antioxidant-neurotrophic axis unique among plant polyphenols (Zhao et al., 2014; Zhang et al., 2022). Furthermore, the use of purified anthocyanins has demonstrated beneficial effects on dyslipidemia and insulin resistance in diabetic patients, further supporting the role of polyphenols in managing diabetes-related complications (Li D. et al., 2015).

### 3.2 Terpenoids

These compounds, including essential oils and carotenoids, are known for their antioxidant activities. These compounds not only reduce oxidative damage but also modulate inflammatory pathways, thereby providing a dual mechanism of action that is beneficial for DN treatment. A review has highlighted the antioxidative potential of terpenoids, emphasizing their ability to scavenge free radicals and enhance the body’s antioxidant defenses. This is particularly relevant in diabetic conditions where elevated levels of ROS can lead to cellular damage and exacerbate neuropathic symptoms (González-Burgos and Gómez-Serranillos, 2012). Terpenoids such as eugenol and trans-anethole have been reported to possess significant antioxidative effects, which can lead to improved glucose metabolism and reduced OS markers in diabetic rats (Kokabiyan et al., 2023; Sheikh et al., 2015). In addition to their antioxidant properties, terpenoids also exhibit anti-inflammatory effects that can be beneficial in managing DN. For example, studies have demonstrated that certain terpenoids can modulate inflammatory pathways, reducing the expression of pro-inflammatory cytokines and promoting a more favorable cytokine balance in the nervous system. This modulation is essential for alleviating the inflammatory processes that contribute to nerve damage in DN (Cheng et al., 2014). Moreover, natural extracts rich in terpenoids, like those from *Hura crepitans*, have been evaluated for their potential in alleviating hyperglycemia and OS (Lukman et al., 2024).

### 3.3 Carotenoids

Carotenoids, a class of natural pigments found in many fruits and vegetables, exhibit strong antioxidant properties that can help mitigate oxidative damage in diabetic patients. They are critical in protecting cells from oxidative damage (Eggersdorfer and Wyss, 2018). Their ability to enhance insulin sensitivity and protect against OS makes them a valuable component in the dietary management of diabetes and its complications, including DN (Roohbakhsh et al., 2017). Astaxanthin, a powerful antioxidant found in marine organisms, has been shown to protect neuronal cells from OS and improve glucose metabolism, which is crucial for individuals suffering from DN. Its multifaceted actions include reducing inflammation and enhancing the antioxidant defense system (Ciapała et al., 2024). In addition, astaxanthin distinguishes itself by inserting into mitochondrial cardiolipin, a membrane-stabilizing action not shared by flavonoids or terpenoids (Wolf et al., 2010). In a study conducted on a Chinese population, serum carotenoids were found to be associated with a reduced risk of diabetes and diabetic retinopathy, another complication related to OS. Specifically, β-carotene was associated with a reduced risk for diabetes, implying the potential protective effects of carotenoids in diabetic conditions (She et al., 2017).

Lycopene, a potent antioxidant, has been found to protect against central and peripheral neuropathy by inhibiting pathways associated with OS and inflammation in diabetic models (Celik et al., 2020). It triggers antioxidant defenses and increase the expression of components that detoxify advanced glycation products in the kidneys of diabetic rats. In combination with insulin, Lycopene could effectively control glycemia and counteract glycOS, thereby mitigating diabetic complications (Figueiredo et al., 2024). In addition, the combination of curcumin and lycopene significantly improved metabolic parameters and reduced OS in diabetic models. This synergistic effect suggests that dietary strategies incorporating these carotenoids could be beneficial in preventing or alleviating DN (Assis et al., 2017).

### 3.4 Vitamins

Vitamins also have potential therapeutic effects in treating DN. Vitamin E, a well-known antioxidant, has been studied for its effects on DN. A meta-analysis of randomized controlled trials (RCT) indicated that vitamin E supplementation might improve nerve conduction velocity in diabetic patients (Huo et al., 2023). Moreover, the intake of dietary antioxidants, particularly vitamin E and selenium, was observed to have protective effects against diabetic retinopathy, a condition related to OS and often associated with DN (She et al., 2021). For mechanism, the antioxidant properties of vitamin E are linked to improvements in serum paraoxonase-1 activity and some metabolic factors in patients with type 2 diabetes, although they did not significantly affect nitrite/nitrate levels. This suggests that vitamin E may enhance antioxidant capacity and improve metabolic health in diabetic patients, potentially benefiting those with neuropathy (Rafraf et al., 2016). Vitamin C has also been investigated for its role in DN. A study examining the effects of vitamins C and E on diabetic rats found that these vitamins could partially restore testosterone levels and reduce the hypersensitivity of the vas deferens, indicating a potential protective effect on the male reproductive system affected by DN (Fernandes et al., 2011). Another study demonstrated that vitamin B3 supplementation in diabetic rats led to improvements in hyperglycemia, OS, and DNA damage (Abdullah et al., 2018).

Alpha-lipoic acid (ALA) is a potent vitamin-like antioxidant which is found naturally in mitochondria, and also synthesized in liver of animals. A recent study indicated that ALA supplementation could prevent the progression of diabetic nephropathy, another complication of diabetes, by exerting anti-inflammatory and antioxidant effects (Dugbartey et al., 2022). Moreover, ALA exerts antioxidant effects via a dual mechanism: (i) rapid redox-cycling between its disulfide (ALA) and dihydrolipoate (DHLA) forms, which directly quenches ROS without transcriptional activation, and (ii) secondary upregulation of the Nrf2 pathway and suppression of NF-κB nuclear translocation (Ghibu et al., 2009). This suggests that ALA may have a broader protective role in diabetic complications beyond neuropathy.

## 4 Mechanisms by which natural products exert their antioxidant effects

Natural products, particularly those with antioxidant properties, have been recognized for their potential in mitigating oxidative damage and improving neurological function in DN. Various mechanisms through which these natural products exert their antioxidant effects include:

### 4.1 Modulation of antioxidant enzyme activity

Natural products can enhance the activity of endogenous antioxidant enzymes, such as superoxide dismutase (SOD), catalase, and glutathione peroxidase. This modulation helps to bolster the body’s natural defense mechanisms against OS. Polyphenols can upregulate the expression of these enzymes, thereby improving cellular resilience to oxidative damage (Mattera et al., 2017). Curcumin prevents oxidative damage in DN and diabetic retinopathy by modulating antioxidant enzyme activities, including SOD and NADPH oxidases, which are critical for detoxifying ROS (Yao B. et al., 2023). Similarly, naringin was reported to alleviate OS and enhance the activity of endogenous antioxidants in streptozotocin-induced DN models, thereby improving behavioral, biochemical, and OS parameters, reducing TNF-α/IL-6, and restoring brain/pancreatic tissue integrity (Ahmad et al., 2022).

### 4.2 Scavenging of ROS

Natural antioxidants, such as polyphenols, flavonoids, and carotenoids, can directly scavenge free radicals and reactive oxygen species, thereby neutralizing their harmful effects. For instance, polyphenols found in fruits and vegetables have been shown to detoxify ROS and preserve antioxidant proteins, contributing to cellular protection against oxidative damage (Metere and Giacomelli, 2017). Flavonoids such as quercetin and naringenin have significant ROS scavenging abilities, which help protect neuronal cells from oxidative damage (Abdelkader et al., 2020; Chen et al., 2015).

### 4.3 Inhibition of inflammation

Natural products often possess anti-inflammatory properties that complement their antioxidant effects. By inhibiting pro-inflammatory cytokines and signaling pathways, these compounds can reduce OS induced by inflammation. For instance, curcumin and resveratrol were shown to modulate inflammatory responses, thereby mitigating oxidative damage in various disease contexts (Hrelia and Angeloni, 2020). Quercetin protects against diabetes-induced exaggerated vasoconstriction and reducing inflammation. It inhibits the NF-κB signaling pathway, which is involved in inflammatory responses, thereby offering protection against vascular complications in diabetes (Mahmoud et al., 2013). Additionally, Quercetin has also been reported to have therapeutic potential in diabetic neuropathy and retinopathy by improving neuronal function and reducing OS and inflammation (Saikia et al., 2024).

### 4.4 Protection of cellular components

Natural antioxidants can protect critical cellular components, including lipids, proteins, and DNA, from oxidative damage, which is vital in preventing the progression of diseases linked to OS, such as cancer and neurodegenerative disorders. For instance, flavonoids are able to stabilize cell membranes and prevent lipid peroxidation, thereby preserving cellular integrity (Vieira Gomes et al., 2019). Compounds such as grape seed proanthocyanidins (GSPs) have demonstrated protective effects against DPN by alleviating endoplasmic reticulum stress and enhancing antioxidant defenses. The administration of GSPs not only improved nerve conduction velocity but also reduced markers of OS in diabetic models (Ding et al., 2014).

### 4.5 Protection of mitochondrial function

Mitochondria are critical for energy production and are also a major source of ROS. Mitochondrial dysfunction in DN is often linked to increased production of ROS, which can lead to oxidative damage and impaired energy metabolism. Chronic hyperglycemia can induce mitochondrial permeability transition, which compromises mitochondrial function and exacerbates cellular injury in diabetic hearts (Sloan et al., 2012). This underscores the importance of maintaining mitochondrial homeostasis to prevent the progression of neuropathic symptoms. Natural antioxidants can protect mitochondrial integrity and function, thereby reducing the production of ROS. For example, resveratrol was found to improve mitochondrial function and reduce OS in diabetic models (Coyoy-Salgado et al., 2019; Moukham et al., 2024). Pinocembrin was revealed to exert neuroprotective effects by enhancing mitochondrial function and reducing oxidative damage in diabetic animal models (Shen et al., 2019). Ciliary neurotrophic factor (CNTF) has also been identified as a protective agent that enhances mitochondrial bioenergetics in sensory neurons during diabetes. CNTF treatment was demonstrated to activate the NF-κB signaling pathway, which is involved in promoting mitochondrial function and preventing neuropathy in diabetic rodents (Saleh et al., 2013).

### 4.6 Regulation of cellular signaling pathways

**FIGURE 2:**
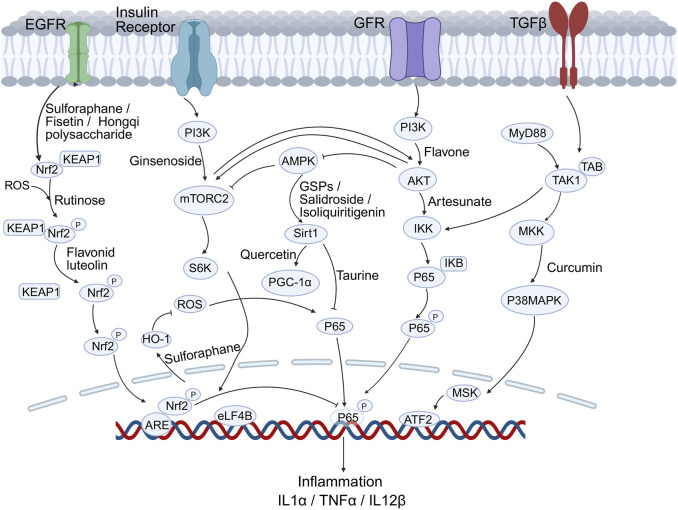
Key signalling cascades modulated by natural antioxidants in DN. Compounds such as Sulforaphane, Fisetin, Quercetin, Curcumin and Taurine activate the Nrf2/Keap1-ARE, PI3K-Akt-mTOR and SIRT1-PGC-1α pathways while inhibiting NF-κB–driven inflammation (IL-1β, TNF-α, IL-12β).

OS in nerve injury and damage is closely linked to several signaling pathways that mediate cellular responses (Figure 2). Natural antioxidants can influence various cellular signaling pathways involved in OS responses. Recent studies have highlighted the role of specific antioxidants in modulating these signaling pathways.

#### 4.6.1 Nrf2/ARE pathway

The Nrf2-antioxidant response element (Nrf2/ARE) pathway is pivotal in counteracting OS, a key driver of DN pathogenesis. Activation of this pathway enhances antioxidant gene expression, offering neuroprotection. In DN, OS is exacerbated by high glucose levels, leading to nerve damage and dysfunction. Activation of the Nrf2/ARE pathway can mitigate these effects by enhancing the body’s antioxidant capacity and reducing inflammation.

Research has shown that various compounds and interventions can activate the Nrf2/ARE pathway, offering potential therapeutic benefits for DN. Sulforaphane, a compound found in cruciferous vegetables, was validated to activate the Nrf2/ARE pathway and inhibit NF-κB pathway, thereby providing antioxidant and anti-inflammatory effects that are beneficial in DN models (Negi et al., 2011b). Similarly, fisetin, a phytoflavonoid, was shown to modulate both the Nrf2 and NF-κB pathways, reducing OS and inflammation (Sandireddy et al., 2016). Melatonin, known for its antioxidant properties, also modulates the Nrf2 pathway, enhancing the expression of heme oxygenase-1 (HO-1), which strengthens antioxidant defenses in DN (Negi et al., 2011a). Luteolin, another flavonoid, has been found to improve nerve functions in DN by upregulating Nrf2 and HO-1 levels, thus enhancing antioxidant capacity and reducing oxidative damage (Li M. et al., 2015). Additionally, hedysarum polysaccharide, derived from *Radix Hedysari*, ameliorated DPN by activating the Keap1/Nrf2 signaling pathway, further supporting the role of Nrf2 activation in managing OS-related complications in diabetes (He et al., 2022).

#### 4.6.2 NF-κB pathway

The NF-κB pathway plays a significant role in the inflammatory processes associated with DN. The interplay between NF-κB and Nrf2 pathways is crucial in managing OS and inflammation, as seen in the effects of dietary phytochemicals that can activate these defense systems (Wu et al., 2022). Apart from fisetin and sulforaphane mentioned above, taurine, an endogenous antioxidant, was shown to alleviate OS in diabetic rats by regulating the NF-κB and Nrf2/HO-1 signaling cascades. This regulation not only reduces inflammation but also promotes neuronal survival and function (Agca et al., 2014). In addition, the TNF-α/NF-κB pathway was also implicated in up-regulating voltage-gated sodium channels, such as Nav1.7, in dorsal root ganglion neurons, contributing to the chronic pain experienced in DN (Huang et al., 2014).

#### 4.6.3 MAPK pathways

MAPK pathways, particularly the p38 MAPK and ERK pathways, are implicated in the inflammatory response associated with DN. Studies have demonstrated that the inhibition of the MAPK/ERK pathway can promote oligodendrocyte generation and recovery in demyelinating diseases, suggesting a potential therapeutic approach for DN (Suo et al., 2019). Compounds such as curcumin, which possess both antioxidant and anti-inflammatory properties, have been investigated for their potential to ameliorate DN by regulating the MAPK signaling pathway (Li et al., 2022).

#### 4.6.4 PI3K/Akt/mTOR pathway

The PI3K/Akt/mTOR signaling pathway plays essential roles in various cellular processes, including growth, proliferation, and survival. The role of the PI3K/Akt/mTOR pathway in DN has been emphasized in a review report, which suggested that targeting this pathway could improve nerve regeneration and prevent degeneration (Pham, 2024). Another research article introduced how taurine ameliorated axonal damage in diabetic rats by activating the PI3K/Akt/mTOR signaling pathway (Zhang M. et al., 2021). Artesunate was shown to inhibit apoptosis and promote survival in Schwann cells via the PI3K/Akt/mTOR axis in DPN (Zhang et al., 2023). Similarly, a study on salidroside demonstrated its ability to ameliorate insulin resistance through activation of the AMPK/PI3K/Akt/GSK3β pathway, implicating its potential in managing diabetic complications (Zheng et al., 2015). Flavonoids derived from citrus fruits exhibit antioxidant properties that can enhance glycemic control and modulate signaling pathways related to insulin sensitivity and OS (Gandhi et al., 2020). They were also found to activate the PI3K/Akt pathway, which promotes cell survival and reduces apoptosis in neuronal cells exposed to OS (Bakhtiari et al., 2017; Ayaz et al., 2019). Recently, a study investigated curcumin’s mechanism in DPN using high glucose-stimulated Schwann cells, revealing that curcumin reduces apoptosis, upregulated Hmox1/Akt signaling, and bound Hmox1 via molecular docking (Lin et al., 2024).

#### 4.6.5 SIRT1/PGC-1α pathway

The Sirtuin 1/PPAR-γ co-activator1α (SIRT1/PGC-1α) pathway is critical in regulating mitochondrial function and OS, which are key factors in the pathogenesis of DN. Several antioxidants have been shown to modulate this pathway. For instance, isoliquiritigenin could reduce oxidative damage and alleviate mitochondrial impairment by activating SIRT1, which in turn enhances the PGC-1α signaling pathway in experimental DN models. This activation leads to improved mitochondrial biogenesis and function, thereby mitigating the neuropathic effects of diabetes (Yerra et al., 2017). Quercetin was demonstrated to correct mitochondrial abnormalities in DPN by activating the AMPK/PGC-1α pathway (Zhang Q. et al., 2021). Moreover, grape seed procyanidin B2 has been reported to protect podocytes from high glucose-induced mitochondrial dysfunction and apoptosis via the AMPK-SIRT1-PGC-1α axis. This protection is crucial in preventing the cellular damage associated with diabetic nephropathy, which might also provide new insights into DN treatment (Huang et al., 2023). In addition, the involvement of the SIRT1/PGC-1α pathway in the protective effects of antioxidants against DN is also evident in studies involving nicotinamide mononucleotide (NMN). NMN administration prevents diabetes-induced cognitive impairment by activating the SIRT1 pathway, which preserves mitochondrial function and reduces OS (Chandrasekaran et al., 2020).

## 5 Clinical application of natural antioxidants in DN

### 5.1 From rodent to human reality

Although animal models have been extensively used in initial functional tests and mechanism research, there are also limitations that are difficult to overcome. Animal studies routinely use body-weight–based dosing that, once scaled to human physiology, can exceed safety limits set for oral consumption. Rodents also enjoy higher intestinal absorption and weaker first-pass metabolism than humans, meaning an identical milligram-per-kilogram dose yields far lower plasma exposure in people. Moreover, the blood–nerve barrier is tighter in humans, so compounds that flood peripheral nerves in rats may barely reach therapeutic thresholds in patients. Finally, rodents develop neuropathy within weeks, whereas human DN unfolds over years, allowing compensatory mechanisms that animal models simply lack. These inherent pharmacokinetic and pathophysiological gaps demand careful human-equivalent dosing and long-term translational studies before antioxidant benefits can be claimed (Reagan-Shaw et al., 2008; Sharma and McNeill, 2009; Blanchard and Smoliga, 2015).

### 5.2 Clinical trials of natural antioxidants in DN

Although rodent models provide mechanistic insights, they often oversimplify the chronic, multifactorial nature of human diabetes. Therefore, translational research must account for these differences to improve clinical relevance. Various clinical trials and studies have explored the efficacy of natural antioxidants, demonstrating promising results in alleviating symptoms and improving nerve function (Table 3).

**TABLE 3:** Description of the antioxidant natural products and pharmacological aspects of the clinical studies included in the review.

Substance	Dose & route (daily)	Patients (n)	Duration	VAS pain Δ (cm)	Sural NCV Δ (m s^−1^)	TCSS Δ (points)	Adverse events (≥1%)	Ref
Anthocyanins	160 mg p.o.	150 T2DM	24 weeks	NR	NR	−1.0	NR	Li et al. (2015a)
Vitamin E	400 IU p.o.	60 T2DM	8 weeks	NR	+1.5	NR	GI upset 3%	Rafraf et al. (2016)
R(+)-thioctic acid	1.6 g p.o.	30 T2DM	30 days	−1.4	+2.2	−1.3	Nausea 7%	Mrakic-Sposta et al. (2018)
Curcumin	80 mg p.o.	40 T2DM	8 weeks	−0.9	+1.6	−1.5	Diarrhea 5%	Asadi et al. (2019)
ALA	600 mg p.o.	181 DPN	6 months	−1.2	+2.1	−1.4	Nausea 6%	El-Nahas et al. (2020)
γ-Linolenic acid	320 mg p.o.	130 DPN	12 weeks	−0.8	+1.7	−1.1	Dyspepsia 4%	Won et al. (2020)
Vitamin B12	1 mg p.o.	90 DPN	12 months	−0.7	+1.8	−1.2	NR	Didangelos et al. (2021)
SOD + ALA + B12 + ALC combo	250 mg p.o.	90 DPN	12 months	−1.0	+1.9	−1.3	Headache 3%	Didangelos et al. (2020)
Melatonin	3–6 mg p.o.	63 DPN	8 weeks	−1.3	NR	−1.0	Somnolence 8%	Shokri et al. (2021)

NR, not reported; Δ, change from baseline.

One notable study investigated the effects of R (+)-thioctic acid, a biogenic antioxidant, on OS and peripheral neuropathy in diabetic patients, indicating significant improvements in OS markers and nerve conduction velocity (Mrakic-Sposta et al., 2018). Curcumin (80 mg/day for 12 weeks) enhanced glycemic control and antioxidant markers (TAC, glutathione) in diabetic foot ulcer patients but failed to accelerate wound healing (Mokhtari et al., 2021). Green tea extract (16-week trial) improved Toronto Clinical Scoring System (TCSS) scores, pain intensity (VAS), and vibration perception threshold (VPT) in mild-to-moderate DPN. Benefits became evident after 8 weeks, with progressive improvements by week 16 (Essm et al., 2021).

A RCT study demonstrated that ALA supplementation significantly alleviated neuropathic symptoms in patients with DPN. In this study, participants receiving ALA showed a marked reduction in the Neuropathy Symptoms Score compared to those who did not receive treatment, the patients treated with ALA reported an average time of 18.4 days for symptom relief (Pieralice et al., 2019). Furthermore, a meta-analysis of multiple studies confirmed that ALA, when combined with other therapies, such as epalrestat, exhibited superior efficacy in improving nerve conduction velocities and reducing neuropathic pain (Zhao et al., 2018). Recently, more clinical studies have explored ALA application in treating DN either alone or combined with other treatments. A 6-month, double-blinded, placebo-controlled trial for DPN patients demonstrated that oral ALA (600 mg twice daily) significantly improved neurological symptom scores (NSS), neurological disability scores (NDS), VAS, and VPT compared to placebo. Mild nausea (6% incidence) was the only reported adverse effect, with no treatment discontinuations (El-Nahas et al., 2020). In a 12-week noninferiority trial comparing γ-linolenic acid (GLA, 320 mg/day) to ALA (600 mg/day), both agents reduced VAS and total symptom scores (TSS), with GLA meeting noninferiority criteria for pain reduction. However, TSS results crossed the noninferiority margin, suggesting ALA may still hold a slight edge (Won et al., 2020).

Vitamin B12 deficiency is common in metformin-treated diabetics, which exacerbates neuropathy. A 1-year randomized trial administering oral methylcobalamin (1 mg/day) to patients with DN and low B12 levels (<400 pmol/L) showed significant improvements in sural nerve conduction velocity (SNCV), amplitude (SNAP), VPT, VAS, and quality of life (QOL), underscoring B12’s neuroprotective role (Didangelos et al., 2021). A combination therapy trial (Superoxide Dismutase, ALA, Acetyl L-Carnitine, and B12) over 12 months further supported B12 and ALA benefits. The active group exhibited improved SNCV, SNAP, VAS, and QOL, though autonomic function (CARTs) remained unaffected (Didangelos et al., 2020).

As an adjunct to pregabalin, melatonin (3–6 mg/day) significantly reduced pain, sleep interference, and improved QOL in an 8-week trial. Over 63% of melatonin-treated patients achieved ≥50% pain reduction, highlighting its potential as an adjuvant therapy (Shokri et al., 2021). Similarly, CoQ10 (100 mg three times daily) combined with pregabalin outperformed placebo in pain reduction and sleep interference after 8 weeks. Responder rates and global improvement were also higher, supporting CoQ10’s anti-inflammatory and antioxidant synergy with standard therapy (Amini et al., 2022).

Across trials that employed identical endpoints mentioned above, antioxidant benefits were consistently modest. For pain intensity (VAS, 0–10 cm), ALA 600 mg/day produced the largest reduction (−1.2 cm, d ≈ 0.40), followed by CoQ10 (−1.0 cm, d ≈ 0.35), green-tea extract (−0.9 cm, d ≈ 0.30) and GLA (−0.8 cm, d ≈ 0.25); melatonin as an adjuvant yielded a responder rate of 64% versus 34% placebo (RR ≈ 1.9). When sural-nerve conduction velocity was the common metric, α-lipoic acid (+2.1 m/s, d ≈ 0.33), vitamin B12 (+1.8 m/s, d ≈ 0.32) and a multi-antioxidant cocktail (+1.9 m/s, d ≈ 0.31) clustered within a narrow 0.3 m/s range. Only curcumin reported TCSS change (−1.5 points, d ≈ 0.35). In Cohen’s terms, all observed effects fall between small and moderate; none approached the thresholds generally considered large in neuropathic pain trials, underscoring the need for larger, longer and better-standardized studies.

In addition to these findings, a systematic review highlighted the effectiveness of anthocyanin-rich foods in improving cardiometabolic factors, which are often compromised in individuals with diabetes. The review concluded that these foods could positively influence markers related to DN, such as lipid profiles and inflammatory markers, further supporting the integration of dietary antioxidants into treatment regimens (Araya-Quintanilla et al., 2023). Moreover, the potential of dietary polyphenols as therapeutic agents for diabetes and its complications has been extensively documented. These compounds, found in various fruits and vegetables, exhibit antioxidant, anti-inflammatory, and neuroprotective properties, making them valuable in the management of DN (Aryal et al., 2024).

Among those antioxidants reviewed, only ALA—backed by nearly 1,800 patients across four RCTs—and nano-curcumin, whose dual NOX4 inhibition and NGF boost has been confirmed in two pilot trials, emerge as the most translation-ready candidates; directing the next wave of adaptive Phase II/III studies to these two agents will most efficiently convert mechanistic promise into proven clinical benefit for DN.

### 5.3 Therapeutic limitations of natural antioxidants

Although numerous antioxidants have been found to be promising in treating DN, these antioxidants often face limitations such as poor solubility and bioavailability, limited absorption and rapid metabolism, which reduce their effectiveness in clinical applications. Natural polyphenolic compounds often face challenges such as low water solubility and first-pass metabolism. For instance, curcumin is hindered by poor oral bioavailability, limiting its therapeutic efficacy and preventing effective doses from reaching therapeutic thresholds in neural tissues (Dwivedi et al., 2022). Resveratrol protects against peripheral neuropathy by modulating mitochondrial dysfunction, but its suboptimal bioavailability restricts its clinical application (Yao Y. et al., 2023).

The extremely low permeability of natural antioxidants across the BNB presents a significant challenge in the treatment of DPN. The BNB is a selectively permeable barrier that protects peripheral nerves by maintaining a controlled microenvironment, similar to the blood-brain barrier (BBB) in the central nervous system. Consequently, the clinical application of natural antioxidants in treating DPN is hindered by their poor permeability across the BNB (Takeshita et al., 2020). Another point is that DN involves multi-pathway interactions related to OS, neuroinflammation, and axonal degeneration. However, single antioxidants struggle to simultaneously regulate key pathways like Keap1/Nrf2, SIRT1/PGC-1α, and PI3K/Akt, leading to restricted therapeutic efficacy.

### 5.4 Strategies to overcome the limitations of natural antioxidants

To address these challenges, innovative strategies such as nanotechnology-based delivery systems have been explored to enhance the therapeutic potential of natural antioxidants in DN treatment. By employing nanocarriers such as liposomes, nanoparticles, and dendrimers, researchers aim to enhance the delivery and bioavailability of antioxidants. These nanocarriers can be engineered to improve the solubility, stability, and targeted delivery of antioxidants to specific tissues, thereby maximizing their therapeutic potential. Application of nanotechnology to enhance the biological activity of herbal antioxidants showed protential in improving the efficacy of these compounds in managing OS-related conditions, without significant side effects (Mishra et al., 2022). In a study focusing on the green synthesis of silver nanoparticles using *Nigella sativa* extract, these nanoparticles exhibited significant anti-inflammatory and antioxidant effects (Alkhalaf et al., 2020). Lipid nanoparticles and polymeric nanoparticles have been explored for their ability to deliver therapeutic agents effectively to ocular tissues in diabetic retinopathy, a condition closely related to DN (Selvaraj et al., 2017). Moreover, the encapsulation of Dipeptidyl peptidase-4 (DPP4)/CD26 in mesoporous silica nanoparticles has demonstrated sustained release properties and enhanced bioavailability, which are crucial for the effective management of hyperglycemia and its complications, including DN (Huang et al., 2017). A clinical study demonstrated that nano-curcumin supplementation for 8 weeks significantly reduced the severity of diabetic sensorimotor polyneuropathy in patients with T2DM, as evidenced by improved neuropathy scores and glycemic control (Asadi et al., 2019). Another RCT study in diabetic patients with compression-resistant leg ulcers found topical quercetin/oleic acid nano-hydrogel significantly accelerated wound healing vs hyaluronic acid, with no adverse effects (Gallelli et al., 2020). In addition, a collagen-laminin matrix impregnated with resveratrol microparticles accelerated diabetic foot ulcer healing by 57.8% vs. 26.6% with standard care. It reduced OS markers (TNF-α, caspase 3) and improved glutathione ratios, demonstrating dual antioxidant and tissue-regenerative effects (Çetinkalp et al., 2021).

Animal and cellular experiments also proved the treating effects and explored the mechanisms of nano-antioxidants in DN treatments. A study found nanoparticle-encapsulated curcumin alleviates diabetic neuropathic pain by targeting P2Y12 receptors on activated DRG satellite glial cells (SGC) in diabetic rats, suppressing IL-1β/Cx43/p-Akt pathways, reducing CGRP expression, and inhibiting SGC-mediated inflammatory responses, thereby improving mechanical/thermal hyperalgesia (Jia et al., 2017). In diabetic rats, an oral sesamol-PLGA nanosuspension was developed for diabetic foot ulcers, leading to reduced TNF-α, upregulated HSP-27/ERK/PDGF-B/VEGF, enhanced collagen deposition and angiogenesis, accelerated re-epithelization, and reduced inflammation, restoring impaired wound healing via anti-inflammatory and pro-regenerative pathways (Gourishetti et al., 2020). A study developed ultrasound-enhanced α-tocopherol (ATF)-liposomes for topical DN treatment. ATF-liposomes reduced ROS in high-glucose-exposed Schwann cells (IMS-32) and penetrated porcine skin effectively with sonication, demonstrating localized antioxidant efficacy (Heo et al., 2025). Another cellular study developed avanafil nanocomplexes using chitosan/zein and antioxidants (α-lipoic/ellagic acids) via Box-Behnken design. In high-glucose PC12 cells, this formulation reduced ROS/lipid peroxidation and enhanced viability, demonstrating improved solubility and neuroprotection (Kurakula et al., 2021).

### 5.5 Translational reality check

Across all antioxidant classes, a recurring triad of contradiction, pharmacokinetic failure, and dosing uncertainty clouds the leap from bench to bedside. Polyphenols such as curcumin elevate nerve-conduction velocity and NGF in rodents (Zhang et al., 2022), yet a 12-week 80 mg/day RCT in diabetic-foot-ulcer patients improved glycaemic control without accelerating wound closure (Mokhtari et al., 2021). The limiting step of curcumin treatment is the low oral bioavailability, with even optimized nano-formulations barely reaching one-tenth of the neuroprotective plasma levels documented in rats (Dwivedi et al., 2022; Anand et al., 2007). Eugenol abolishes hyperalgesia at 25 mg kg^−1^ or more in rats (Kokabiyan et al., 2023), whereas an appropriately powered human study with comparable eugenol dosing has not yet been reported, leaving the clinical effect on NCV or other electrophysiological parameters unknown. Carotenoids display similar dissonance, which rescue sciatic nerves at 45 mg kg^−1^ lycopene (Figueiredo et al., 2024) yet human plasma saturates at ∼1 µM after 30 mg oral intake—over 30-fold below rodent neuroprotective exposure—and optimal human dosing remains undefined (Maiani et al., 2009). Marine astaxanthin reduces pain of diabetic neuropathy at 50 mg kg^−1^ in rats, but no RCT trials have reported for diabetic neuropathy pain (Ciapała et al., 2024). Finally, ALA exemplifies the dosing dilemma: 600 mg/day benefits neuropathic symptoms, yet 100 mg/day failed, while intravenous injection offers 4-fold higher exposure at the cost of clinical infrastructure (El-Nahas et al., 2020; Ziegler et al., 1995). Together, these realities underscore the imperative for standardized endpoints, precise human Pharmacokinetics–Pharmacodynamics mapping, and scalable nanocarrier strategies before antioxidant therapy can claim genuine clinical efficacy for DN.

### 5.6 Limitations of the clinical research

Although several natural antioxidants have been evaluated in human studies of DN, the available clinical evidence is limited by a cluster of recurring methodological weaknesses that restrict the ability to draw firm conclusions.

#### 5.6.1 Sample size and statistical power

Most trials are single-center studies with small sample sizes (Table 2). Among the 12 RCTs reviewed, only three enrolled >150 participants (El-Nahas et al., 2020; Won et al., 2020; Didangelos et al., 2020). Post-hoc power calculations (G*Power 3.1) using the reported effect sizes (Cohen’s d 0.25–0.40) indicate that ≥80% power to detect a minimal clinically important difference of 1 cm on the VAS pain scale would require 128–200 completers per arm; only two studies met or approached this threshold (El-Nahas et al., 2020; Didangelos et al., 2020). Consequently, the risk of type II error is high, and negative findings must be interpreted cautiously.

#### 5.6.2 Study design and blinding

Although most trials claim double-blinding, only four explicitly report allocation concealment and assess blinding integrity via participant/assessor guess questionnaires (Mokhtari et al., 2021; El-Nahas et al., 2020; Didangelos et al., 2021; Shokri et al., 2021). Moreover, the absence of active-placebo controls in polyphenol or vitamin trials raises the possibility of performance bias, particularly when subjective endpoints such as VAS are used (Pop-Busui et al., 2017).

#### 5.6.3 Heterogeneity of patient populations

Baseline heterogeneity is pronounced. Mean diabetes duration ranges from 5.2 ± 2.8 years (Mokhtari et al., 2021) to 15.3 ± 4.1 years (El-Nahas et al., 2020), HbA1c from 7.1% (Essm et al., 2021) to 9.4% (Didangelos et al., 2020), and concomitant medications vary widely (metformin, insulin, SGLT2 inhibitors, pregabalin).

#### 5.6.4 Endpoint selection and standardization

Across the 12 RCTs, seven different primary endpoints were used (VAS, TCSS, NDS, VPT, NSS, SNAP, SNCV), and only two trials adopted composite scores recommended by the Toronto Consensus on Diabetic Neuropathy (Pop-Busui et al., 2017). Furthermore, only four studies reported MCID thresholds, and none performed responder analyses beyond the ≥50% VAS reduction criterion (Shokri et al., 2021; Amini et al., 2022). The lack of harmonization hampers meta-analytical synthesis and cross-study comparison.

#### 5.6.5 Follow-up duration and attrition

Median follow-up is 12 weeks (range 4–52 weeks), whereas progression/regression of DN typically manifests over years. Attrition exceeded 15% in three trials (Mokhtari et al., 2021; Won et al., 2020; Didangelos et al., 2021), with differential dropout between antioxidant and placebo arms (e.g., 18% vs 6% for curcumin (Mokhtari et al., 2021)), introducing potential attrition bias.

#### 5.6.6 Safety and tolerability reporting

Adverse events were reported in only eight trials, and none employed the NIH Common Terminology Criteria. Gastrointestinal events occurred at 3%–7% incidence with ALA and curcumin, but no trial was powered to rule out rare but clinically relevant toxicities. Despite the mechanistic appeal of natural antioxidants, their adverse-effect and long-term safety profiles remain under-reported and under-analysed, representing a major translational barrier.

In summary, while the reviewed clinical studies provide encouraging signals, they suffer from small samples, heterogeneous populations, non-standardized endpoints, and short durations. Future RCTs should adopt the NIH–NINDS Common Data Elements for DN, pre-specify subgroup analyses based on glycaemic control and medication use, and employ adaptive or platform designs to achieve adequate power while accounting for heterogeneity.

## 6 Future directions and unmet needs

Despite the promising nature of antioxidant therapies, several unmet needs and future directions remain critical for advancing treatment strategies. One of the primary challenges in utilizing antioxidants for DN is the need for effective delivery systems that ensure adequate bioavailability at the target sites. Current formulations often suffer from poor solubility and stability, limiting their therapeutic efficacy. Advanced drug delivery strategies, such as nanomedicine, could enhance the targeting and sustained release of antioxidants, thereby improving their effectiveness in clinical settings. Recent studies have highlighted the potential of nanomedicine-based approaches to specifically target neuroimmune interactions in DN, offering a promising avenue for future research (Balogh et al., 2022). Moreover, the complexity of DN necessitates a multifaceted approach to treatment. While antioxidants are useful in reducing OS, they should be integrated into broader therapeutic regimens that include neuroprotective agents and lifestyle modifications. For instance, dietary interventions that enhance the intake of natural antioxidants, such as polyphenols, could complement pharmacological treatments. Research has shown that polyphenolic compounds can suppress indicators of OS and inflammation, which are prevalent in DPN (Krstić et al., 2021). Therefore, future studies should focus on the synergistic effects of combining antioxidants with other therapeutic modalities.

In addition to improving delivery and combination strategies, there is a pressing need for well-designed clinical trials to evaluate the efficacy of antioxidant therapies in DN. Many existing studies are limited by small sample sizes or lack of standardized outcome measures. Future research should prioritize large-scale, multicenter trials that assess not only the clinical outcomes but also the underlying mechanisms of action of antioxidants in the context of DN. Lastly, ongoing research into the molecular pathways involved in DN will be crucial for identifying new therapeutic targets. Understanding how OS interacts with other pathological processes, such as neuroinflammation and metabolic dysregulation, could lead to the development of novel antioxidant compounds that are more effective in treating DN. This integrative approach could pave the way for innovative therapies that address the multifactorial nature of DN.

## 7 Conclusion

This review underscores the central role of OS in the pathogenesis of diabetic neuropathy (DN) and highlights the therapeutic application of natural antioxidants. Polyphenols, terpenoids, carotenoids, marine and herbal products, as well as vitamins and animal-derived molecules, can counteract oxidative damage, modulate inflammatory cascades, and restore nerve conduction and pain. Mechanistically, these agents activate the Nrf2/ARE, NF-κB, MAPK, PI3K/Akt/mTOR, and SIRT1/PGC-1α pathways, protect mitochondria and the blood–nerve barrier, and upregulate endogenous antioxidant enzymes. Clinical evidence—particularly for α-lipoic acid, curcumin, green tea, vitamin B12, melatonin, and CoQ10—supports symptomatic relief and functional improvement, although trials remain small and heterogeneous. Key limitations include poor bioavailability, rapid metabolism, and restricted penetration across the blood–nerve barrier; emerging nanotechnology-based delivery systems offer potential solutions. Future research should prioritize large-scale, standardized trials, combination strategies, and refined formulations to translate antioxidant benefits into effective, integrative therapies for DN.
